# Effects of Orientations and Regions on Performance of Online Soluble Solids Content Prediction Models Based on Near-Infrared Spectroscopy for Peaches

**DOI:** 10.3390/foods11101502

**Published:** 2022-05-21

**Authors:** Sanqing Liu, Wenqian Huang, Lin Lin, Shuxiang Fan

**Affiliations:** 1 College of Mechanical Engineering, Guangxi University, Nanning 530004, China; lsq0101@163.com (S.L.); huangwq@nercita.org.cn (W.H.); 2 Intelligent Equipment Research Center, Beijing Academy of Agriculture and Forestry Sciences, Beijing 100097, China

**Keywords:** nondestructive detection, rapid detection, full transmittance spectra, multipoint sampling, zone combination method, nectarine

## Abstract

Predicting the soluble solid content (SSC) of peaches based on visible/near infrared spectroscopy has attracted widespread attention. Due to the anisotropic structure of peach fruit, spectra collected from different orientations and regions of peach fruit will bring variations in the performance of SSC prediction models. In this study, the effects of spectra collection orientations and regions on online SSC prediction models for peaches were investigated. Full transmittance spectra were collected in two orientations: stem-calyx axis vertical (Orientation1) and stem-calyx axis horizontal (Orientation2). A partial least squares (PLS) method was used to evaluate the spectra collected in the two orientations. Then, each peach fruit was divided into three parts. PLS was used to evaluate the corresponding spectra of combinations of these three parts. Finally, effective wavelengths were selected using the successive projections algorithm (SPA) and competitive adaptive reweighted sampling (CARS). Both orientations were ideal for spectra acquisition. Regions without peach pit were ideal for modeling, and the effective wavelengths selected by the SPA led to better performance. The correlation coefficient and root mean square error of validation of the optimal models were 0.90 and 0.65%, respectively, indicating that the optimal model has potential for online prediction of peach SSC.

## 1. Introduction

Peach is in favor with customers for its rich nutrition. Flavor of peach is the decisive factor that has direct effects on acceptance of market and customers’ willingness to pay. Soluble solid content (SSC) is one of the factors that influence the flavor of peach, and it is the most commonly used criterion for assessing the flavor. Traditional SSC tests are destructive, time consuming and complex [1]. Hence, advanced SSC detection technologies with noninvasive manners for peach fruit are needed to realize massive and industrial detection.

Nondestructive detection can be applied to industrial fruit sorting as an ideal technology, meeting the demands for being rapid, efficient and simple [2]. It detects quality utilizing the optical, electromagnetic, acoustic, and chemical properties of fruits. There is a series of nondestructive detection methods, such as image processing, near-infrared spectroscopy, magnetic resonance imaging, ultrasonic methods, and electronic nose. The most frequently used method is near-infrared (NIR) spectroscopy. It detects the internal and external qualities of fruit by optical waves whose wavelengths are located in the near-infrared region (780–2500 nm) of electromagnetic spectra. NIR spectroscopy is used to study the absorption, emission, scattering, reflection, and diffuse-reflection properties of materials [3]. Absorption spectra appear frequently in the research on fruit quality. Researchers usually establish relationships between absorption spectra and fruit quality by methods of chemometrics.

Researchers on SSC prediction based on NIR spectroscopy have achieved great success with different fruits such as blueberry [4], pear [5], banana [6], durian [7], and grape [8]. Increasing researchers have turned their focus to the factors affecting the performance of SSC prediction models. The robustness of an SSC prediction model was discussed using samples from different cultivation environments [9]. Liu et al. [10] applied nonlinear modeling and achieved higher accuracy when compared with linear modeling. However, the performance of SSC prediction models was affected by spectra collection orientations and regions, and previous investigators did not consider these kinds of effects. Orientation is the direction in which a selected part of the fruit is facing. There are variances in spectra collected from different orientations. Similarly, there are also variances in spectra collected from different regions of the fruit. Some research on predicting SSC in pear [11] and apple [12] concluded that performance of SSC prediction models was affected by the spectra collection orientations. Nevertheless, according to the final results of both works above, the optimal spectra collection orientations were different between [11,12]. Hence the optimal spectra collection orientation needs specific discussion according to specific fruits. In addition, some researchers suggested that fruit had a natural asymmetric structure and uneven SSC distribution [13,14,15], resulting in variances in spectra from different collection regions. Some related works confirmed this conclusion by comparing the SSC prediction models built by spectra from different collection regions [16,17]. Online detection automatically predicts the SSC of fruits on a working grading line without the involvement of manual work. The fruits move on the grading line in the process of online detection. For online prediction of fruit SSC, considering spectra collection orientations and regions is helpful and necessary but seldom discussed.

Online SSC prediction for fruits based on NIR spectroscopy generally applies reflectance and transmittance modes [18]. However, original spectra in reflectance mode are affected by stray light, and only superficial information on fruits can be acquired under reflectance mode. These disadvantages decrease the accuracy of online SSC prediction models. On the contrary, transmission mode has the advantages of collecting the internal fruit information and higher prediction accuracy in comparison with reflectance mode. Research indicated that transmission mode was proper for online SSC prediction [19]. Full transmission is one of the transmission modes. All of the internal information on fruit can be acquired under full transmission mode, and part of the internal information on fruit can be acquired under semi-transmission mode. Some studies have achieved excellent results for online SSC detection with full transmission spectra [17,20]. Therefore, full transmission spectra have promising potential for online SSC prediction.

State-of-the-art studies about prediction of peach fruit SSC have proposed feasible methods and considered key factors concerning prediction accuracy. Li et al. proposed a modeling method combining different regions of interest (ROI) in hyperspectral images to develop SSC prediction models [1]. Nascimento et al. considered the effects of harvest season and developed a robust SSC prediction model based on NIR spectroscopy to avoid the influence of harvest season [21]. Effects of preharvest factors were discussed for prediction of peach fruit SSC [22]. Minas et al. added new variables (crop load and canopy position during growth) to develop accurate an internal quality prediction model using NIR spectroscopy [23]. Additionally, light absorption and reduced scattering coefficients were also used to predict the SSC of peach fruit [24]. However, online SSC prediction, which is meaningful for practical application, was rarely discussed. Liu et al. [25] developed an online SSC prediction model for peach fruit under different storage temperatures, but their work did not consider the effects of spectra collection orientations and regions.

Previous studies concerned with SSC prediction for peach fruit considered the effects of preharvest factors, postharvest factors, the optical properties of the peach fruit, etc. However, few studies took the influences of spectra collection orientations and regions into consideration in online SSC prediction for peach fruit and also rarely applied full transmission mode. Hence, the objectives of this study were: (1) to collect the original full transmission spectra in different spectra collection orientations and measure SSC values; (2) to build SSC prediction models using the final mean spectra of different spectra collection orientations and compare the performances of the built models; (3) to divide the original spectra of every single sample into three parts that corresponded to different parts of the sample and build SSC prediction models using the final mean spectra of the different combinations of parts for comparison; and (4) to select the effective wavelengths from the final mean spectra of optimal combined parts for SSC prediction and develop the optimal online SSC prediction models for peach fruit.

## 2. Materials and Methods

### 2.1. Samples

A total of 150 Aoyou peaches were collected on 17 July 2021, from Pinggu, Beijing, China. All of the peach fruits were bagging nectarine. The samples were collected from different positions in the canopies of different peach trees. A total of 110 individual collected samples were mature, and the rest were immature. The two levels of maturity were distinct enough to be distinguished by naked eyes; peach fruits with different levels of maturity contain variability that drives a wide range of SSC. Then, the samples were numbered individually. In this study, peach fruit samples were randomly assigned into a calibration set or a validation set. A total of 100 samples were assigned to the calibration set, and the remaining 50 samples were assigned to the validation set.

### 2.2. Spectrum Collection

**Figure 1:**
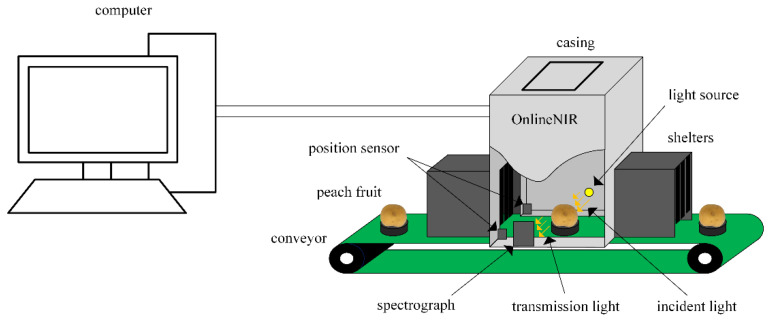
Diagrammatic sketch of the full-transmittance spectrum scanning system.

The NIR spectra of samples were collected by a full-transmittance spectrum scanning system. A sketch of the system is shown in Figure 1. The system consisted of 6 components: (1) a motor-driven conveyor belt that was used to load the peach fruits continuously for online SSC detection; (2) a frame consisting of a casing and shelters that was used to fix the other devices of the system and shield the interference of stray light; (3) a halogen lamp (FUJI, JCR, 150 W, 15 V) that was used as light source; (4) a high-sensitivity spectrometer (Imes 10, NIRECO Corporation, Tokyo, Japan) with a spectral range of 560–1071.75 nm (each spectrum was measured with 0.25 nm intervals); (5) a position sensor that consisted of a photoelectric switch that was used to detect the passing peach fruits and launch the collection of spectra; and (6) a computer that was used to control the system and store the spectral data.

**Figure 2:**
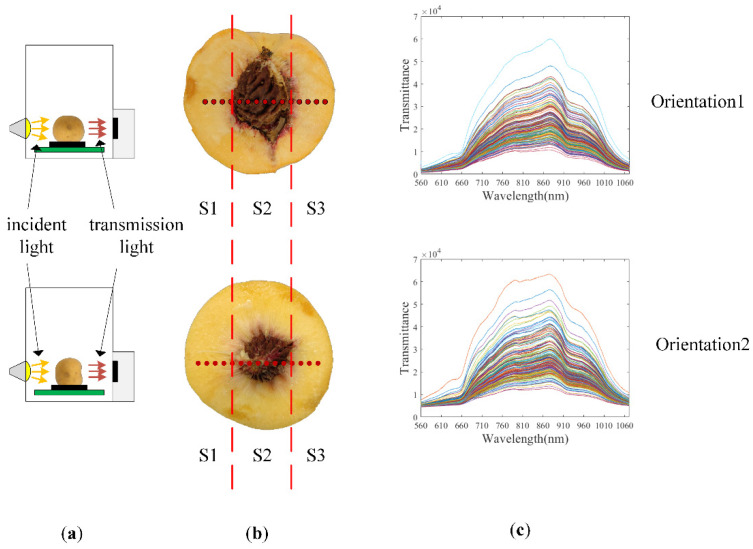
Sketches of the spectra-collecting process in the two orientations for a single sample. Sketch of the placement of samples are shown in the leftmost column (**a**). Sketch of spectra measurement areas (red points) are shown in the middle column, where the red dashed lines are the sketch of the division mode of the peach fruit (**b**). Multiple original transmittance spectra are shown in the rightmost column (**c**) with different colors.

In this study, the effects of two fruit orientations (Orientation1 and Orientation2) were discussed, and the sketches of the two modes are shown in Figure 2.

Orientation1: fruit stem-calyx axis vertical; irradiated from the equator position by the halogen lamp and detected from the opposite equator position by the spectrometer; fruit stem is facing the ground.

Orientation2: fruit stem-calyx axis horizontal; irradiated from the calyx position by the halogen lamp and detected from the stem by the spectrometer; fruit stem is facing the spectrometer.

The spectra of peach fruit were measured on the two different orientations. Each sample was manually placed on a fruit cup. Then, the fruit cup with the sample in it was placed on the conveyor belt. The belt moved continuously at a velocity of 480 mm/s. When the front end of the sample reached the spectrometer, the incident light passed through the sample. The incident light that passed through the sample was received by the spectrometer as an original transmittance spectrum of the single sample ($T_{\mathrm{raw}})$ in a contact-free manner. The integration time of $T_{\mathrm{raw}}$ was 5 ms. As the sample passed through the spectrometer continuously, a series of $T_{\mathrm{raw}}$ was recorded one by one at intervals of 5 ms. As a result, each sample had multiple corresponding $T_{\mathrm{raw}}$, shown in Figure 2b. The number of $T_{\mathrm{raw}}$ was determined by sample diameter. The start and the end of the spectra acquisition were determined by the position sensor. Once the front end of the sample reached the position sensor, a trigger signal was sent to the system, and when the back end of the sample left the position sensor, another trigger signal was sent to the system. Combined with the speed of the conveyer belt, the system knew when to start the spectra acquisition and when to end. The sketches of spectra collecting process in the two orientations are shown in Figure 2.

### 2.3. SSC Measurement

A conventional destructive method was applied to determine the SSC after the spectrum collection. For each sample, all the flesh of the peach fruit was cut off. Then the flesh was wrapped in a piece of gauze and fed into a juicer to extract juice. The squeezed juice was deposited into a clean beaker and shaken well. The next step was dripping droplets of the juice on a digital refractometer (PAL-1, ATAGO, Tokyo, Japan, resolution 0.1%, accuracy ±0.2%) to measure SSC value.

### 2.4. Spectra Preprocessing

It is necessary to preprocess the spectra for a reliable, accurate and stable SSC prediction model. Moving average smooth is a common technique used in the preprocessing of spectra. It can reflect the long-term trends of sequences and eliminate the random fluctuation of sequences. Standard normal variate (SNV) transform is one of the most common normalization techniques in NIR spectroscopy analyses [26]. It is an efficient approach for eliminating the interferences of scatter and particle size [27]. Formula (1) illustrates the process of SNV in detail:

(1) $$T_{\mathrm{SNV}}=\frac{T-\mu }{\sigma }$$

where T represents the spectrum that need to be preprocessed; μ is the mean of T; σ is the standard deviation of T; $T_{\mathrm{SNV}}$ represents the spectrum that was processed by SNV.

In this paper, the original transmittance spectra of each sample were first preprocessed by method 1 for comparison of spectrum collection orientations.

In method 1, the original transmittance spectra of each sample were first averaged to calculate the mean spectrum. Then, the mean spectrum of each sample was truncated for denoising, and the part in the range of 652.25–1026.25 nm was retained. After truncation, the retained part was first preprocessed by moving average smooth with the window length of 9, followed by the preprocessing of SNV. At last, the final mean spectrum of each sample was obtained.

Second, the original transmittance spectra of each sample were preprocessed by method 2 for comparison of spectrum-collection regions.

In method 2, each peach fruit was artificially divided into three approximately equal-width parts along the equator. One of the three parts contains the peach pit and the other two parts do not contain the peach pit. The part that contains the peach pit was named S2. The other parts were named S1 and S3. A sketch of this division mode was shown in Figure 2b. Then, the three parts of the peach fruit were used to form different combinations. The specific combinations were S1 and S3 (S1–S3), S2 (S2), S1 and S2, and S3 (S1-S2-S3). The last combination S1-S2-S3 was the whole peach fruit.

Correspondingly, the original transmittance spectra of each sample were also divided into three parts that corresponded to S1, S2, and S3. The number of spectra for each part was calculated by Formulas (2) and (3):

(2) $$N_{S1}{=N}_{S3}=\left[\frac{N_{\mathrm{raw}}}{3}\right]$$

(3) $$N_{S2}{=N}_{\mathrm{raw}}-N_{S1}-N_{S3}$$

where $N_{\mathrm{raw}}$ represents the number of original transmittance spectra for each sample; $N_{S1}$ represents the number of original transmittance spectra corresponding to S1; $N_{S2}$ represents the number of original transmittance spectra corresponding to S2; $N_{S3}$ represents the number of original transmittance spectra corresponding to S3; and $\left[\frac{N_{\mathrm{raw}}}{3}\right]$ means applying the round up function to the result of $\frac{N_{\mathrm{raw}}}{3}$.

Then, the mean spectra of each combination case were calculated using the three parts of the original transmittance spectra. For example, for calculating the mean spectrum of S1–S3 for a single sample, the original transmittance spectra that corresponded to S1 and S3 of the single sample were averaged. Afterward, all the mean spectra for each combination case were truncated, and the parts in the range of 652.25–1026.25 nm were retained. After truncation, the retained parts were first preprocessed by moving average smooth with the window length of 9, followed by the preprocessing of SNV. At last, the final mean spectra of each combination case were obtained.

### 2.5. PLS and Model Evaluation

Partial least squares (PLS) is one of the conventional statistical methods for NIR spectroscopy analysis. It is used for building the relationships between measured sample spectra and measured sample indexes [28]. PLS uses a designed new space that has lower dimensions to approximate the response-related space of the measured sample spectra [29]. The response-related space of the measured sample spectra is related to the measured sample indexes. The designed new space consists of latent variables (LVs). The LVs are determined iteratively using the measured sample indexes and the measured sample spectra. In addition, PLS is more robust in comparison with multiple linear regression and the principal component regression methods.

In this paper, the original transmittance spectra collected from Orientation1 and Orientation2 were first preprocessed by method 1 for the comparison of the spectrum-collection orientations. For the preprocessed original transmittance spectra at each orientation, the calibration set was used for building the peach fruit SSC prediction models by PLS, and the validation set was used to validate the models for each orientation. The results of calibration and validation for each orientation were compared to determine the best spectrum-collection orientation.

Second, the original transmittance spectra collected from Orientation1 and Orientation2 were preprocessed by method 2 for the comparison of the spectrum-collection regions. For the preprocessed original transmittance spectra of each combination case at each orientation, the calibration set was used for building the peach fruit SSC prediction models by PLS, and the validation set was used to validate these models. The results of calibration and validation for each combination case at each orientation were compared to determine the optimal spectrum-collection regions.

In the process of modeling by PLS, cross-validation was applied and implemented 10 times in order to determine the optimal number of LVs and the optimal model base on the minimum root mean square error of the 10-fold cross-validation (RMSECV). The maximum number of LVs was limited to 20. The determined optimal model was the same SSC prediction model as that from the PLS modeling. The results show the minimum RMSECV values accompanied by the corresponding correlation coefficient $R_{\mathrm{CV}}$.

The PLS regression was implemented in Matlab2019a with libPLS toolbox, available at http://www.libpls.net/ (accessed on 24 February 2022).

In this study, the performance of SSC prediction models was evaluated by the following evaluating indicators: the correlation coefficient (R) and the root mean square error (RMSE). The calculations of R and RMSE are defined by Formulas (4) and (5):

(4) $$R=\frac{\sum_{i=1}^{n}\left(y_{\mathrm{mi}}-{\bar{y}}_{m})(y_{\mathrm{pi}}-{\bar{y}}_{p}\right)}{\sqrt{\sum_{i=1}^{n}{\left(y_{\mathrm{mi}}-{\bar{y}}_{m}\right)}^{2}}\sqrt{\sum_{i=1}^{n}{\left(y_{\mathrm{pi}}-{\bar{y}}_{p}\right)}^{2}}}$$

(5) $$\mathrm{RMSE}=\sqrt{\frac{1}{n}\overset{n}{\underset{i=1}{\sum }}{\left(y_{\mathrm{pi}}-y_{\mathrm{mi}}\right)}^{2}}$$

where $y_{\mathrm{pi}}$ is the predicted SSC of sample i; $y_{\mathrm{mi}}$ is the measured SSC of sample i; ${\bar{y}}_{m}$ and ${\bar{y}}_{p}$ are the mean measured SSC and predicted SSC, respectively; and n is the number of samples that were used for model evaluation. A good model should have a higher R and a lower RMSE.

When evaluating the performance of the model in this study, the evaluating indicators of the calibration set ($R_{C}$, RMSEC) and the validation set ($R_{P}$, RMSEP) were considered in total, as was the optimal number of LVs.

### 2.6. Effective Wavelength Selection

Eliminating the redundant information and collinear variables in the spectra we used for the modeling required effective wavelength selection. In addition, effective wavelength selection would also simplify the calculation of calibration and validation. The successive projections algorithm (SPA) and competitive adaptive reweighted sampling (CARS) are widely applied effective wavelength selection algorithms in NIR spectra analyses. The latter is always combined with PLS regression in practical applications and called CARS-PLS.

SPA applies projection operations and forward selection to acquire subsets of variables that have the minimum collinearity. SPA is appropriate for analyzing NIR spectroscopy with high collinearity [30,31]. CARS has the characteristic that it can extract statistical information from large quantities of sub-models. It is a general strategy that can be used for data analyses in other fields, such as genomic, proteomic and metabolomic studies [30,32].

In this study, CARS and SPA were applied to select the effective wavelengths when the optimal spectrum-collection regions were determined. Then, the selected wavelengths were used for modeling, evaluation, and comparison. Finally, according to the results of evaluation and comparison, the optimal online SSC prediction model for peach fruit was defined.

## 3. Results and Discussion

### 3.1. Original Full Transmittance Spectra and Measured SSC

The full transmittance spectra of a single sample are shown in Figure 2. These spectra were collected in Orientation1 and Orientation2. Though the trends of the spectra collected in Orientation1 and Orientation2 are similar, in the range of 560–1071.75 nm, it can be seen that there are obvious differences between the spectra collected at the two different orientations. The peak of the spectra collected in Orientation2 was higher and more obvious at around 760 nm. The measured SSC values for the calibration set and the validation set are shown in Table 1.

### 3.2. Result of Preprocessing

**Figure 3:**
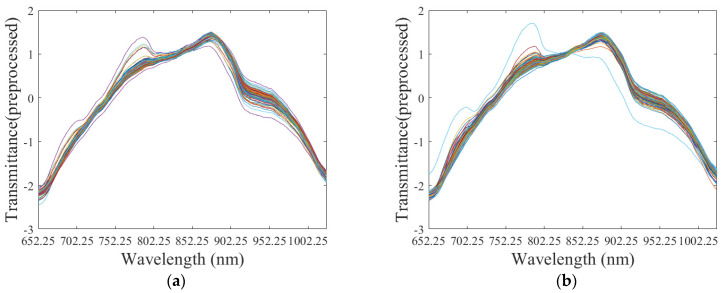
All of the final mean spectra of the S1-S2-S3 combination at Orientation1 (**a**) and Orientation2 (**b**) are displayed with different colors.

All of the final mean spectra for the S1-S2-S3 combination, namely, the original transmittance spectra for each sample preprocessed by method 1, are shown in Figure 3. It can be observed that all spectra have the same trend.

### 3.3. Results of Comparing Spectrum-Collection Orientations and Regions

The results for evaluating the models to predict SSC, built based on the final mean spectra at the different spectrum-collection orientations and different combinations of spectrum-collection regions, are presented in Table 2.

For S1-S2-S3, the model built by preprocessed the spectra at Orientation1 displayed better predictive capacity ($R_{C}$ = 0.93, RMSEC = 0.55%, $R_{P}$ = 0.89, RMSEP = 0.69% for Orientation1 versus $R_{C}$ = 0.95, RMSEC = 0.48%, $R_{P}$ = 0.89, RMSEP = 0.67% for Orientation2). Less difference can be found between the evaluation indicators for Orientation1 and Orientation2. For S1-S3 and S2, the models built with the spectra at Orientation1 have approximate performance to that of the models built with the spectra at Orientation2. Hence, both Orientation1 and Orientation2 are suitable spectral collection orientations for SSC prediction. This conclusion is different from the works about online prediction of apple SSC [17,18]. This can be attributed to differences in fruit structure and composition, as well as in the sizes of seeds. Hence, it is essential to discuss the effects of spectrum-collection orientations for different fruit varieties.

For Orientation1, the model built with the preprocessed spectra of S1-S3 displayed the greatest predictive capacity ($R_{C}$ = 0.93, RMSEC = 0.56%, $R_{P}$ = 0.90, RMSEP = 0.65%), with results similar to those provided by the model built with the preprocessed spectra of S1-S2-S3 ($R_{C}$ = 0.93, RMSEC = 0.55%, $R_{P}$ = 0.89, RMSEP = 0.69%), and the model built with the preprocessed spectra for S2 displayed the lowest predictive capacity ($R_{C}$ = 0.96, RMSEC = 0.43%, $R_{P}$ = 0.78, RMSEP = 0.92%). For Orientation2, there are similar results. The models built with the preprocessed spectra of S1-S2-S3 and S1–S3 have better and similar performance, and the model built with the preprocessed spectra of S2 was unsatisfactory. In general, the spectra collected from the region that contains peach pit have few effects on the performance of the online SSC prediction model. Spectra collected from regions that contain peach pit were not applicable to the modeling. Meanwhile, flesh tissue such as S1–S3 dominates the performance of the model. Therefore, S1–S3 is the optimal combination for SSC prediction.

The spectrum-collection region in peach fruit also has effects on the performance of the online SSC prediction model. According to the results, the spectra collected from S1–S3 are suitable for modeling. This may be caused by the structure of anisotropy. The penetration capability of the light in fruit largely relies on the chemical composition and structural properties of the fruit tissue. Spectra collected from the region that contains peach pit have higher scattering and less information related to SSC, which is supported by related works [17,33]. Therefore, it is an efficient way to collect spectra from the regions that does not contains peach pit for building online SSC prediction models.

In addition, it is remarkably effective to apply full transmittance mode to collecting spectra of fruit using an online system because spectra containing more information related to internal quality of fruit can be collected in full transmittance mode. Additionally, applying full transmittance mode can also simplify the loading mechanism, revealing its promising potential for online detection.

Briefly, in comparison with current studies about online prediction of SSC in peach fruit [25] (Rp = 0.819, RMSEP = 0.841%), this paper took spectrum-collection orientation and region into consideration. Hence, the modeling method applied in this paper was more comprehensive, and the accuracy of our model was higher. This method achieved success in our previous work with apple, which belongs to pome [17], and the feasibility of applying it to peach, which belongs to drupe, has been confirmed in this paper. Hence, we have reason to infer that the method has the potential for extension to other fruits such as pear, plum, cherry, and apricot. Minas et al. [23] proposed multivariate NIRS-based prediction models that used a handheld NIRS sensor. The developed models could estimate the SSC of peach fruit accurately (Rp = 0.96, RMSEP = 0.58%), but they could not be used for massive online detection. Nascimento et al. [21] discussed the effects of maturity stage and harvest season on the prediction models of peach fruit. However, the results were not ideal (Rp = 0.45, RMSEP = 1.04%), and the developed models also could not be used for online detection. Li et al. [1] selected wavelengths in the range of 700–1000 nm for SSC prediction of peach fruit, and a similar range of selected wavelengths was applied in this paper (700–1000 nm). Li et al., Nascimento et al. [1,21] and Liu et al. [25] all adopted reflectance mode and diffuse transmittance mode for spectra collection. In comparison, this paper applied full transmittance mode to acquire the spectra. More internal information on the peach fruit can be acquired in full transmittance mode. Hence, the accuracy of prediction in full transmittance mode was higher than that in reflectance mode or diffuse transmittance mode. In summary, the effects of spectrum-collection orientations and regions on an online SSC prediction model were considered in this study, and the developed models can be used for online SSC prediction of peach fruit.

### 3.4. Effective Wavelength Selection

After the optimal spectra-collection regions were determined, CARS and SPA algorithms were applied for selecting effective wavelengths. The final mean spectra of S1-S3 collected from both Orientation1 and Orientation2 were used for effective wavelength selection, modeling, and evaluation.

SPA selected 10 wavelengths for both Orientation1 and Orientation2, and CARS selected 38 wavelengths and 18 wavelengths, respectively, for the two orientations. Specific selected effective wavelengths are shown in Table 3. The selected wavelengths were mainly distributed in the range of 700–1000 nm, where absorbance was mainly related to the second and third overtones of oxygen-hydrogen (O-H) stretching, and the third and fourth overtones of carbon-hydrogen (C-H) stretching of the organic molecules. Other works also suggested that SSC was related to the absorbance in this range [34,35].

**Figure 4:**
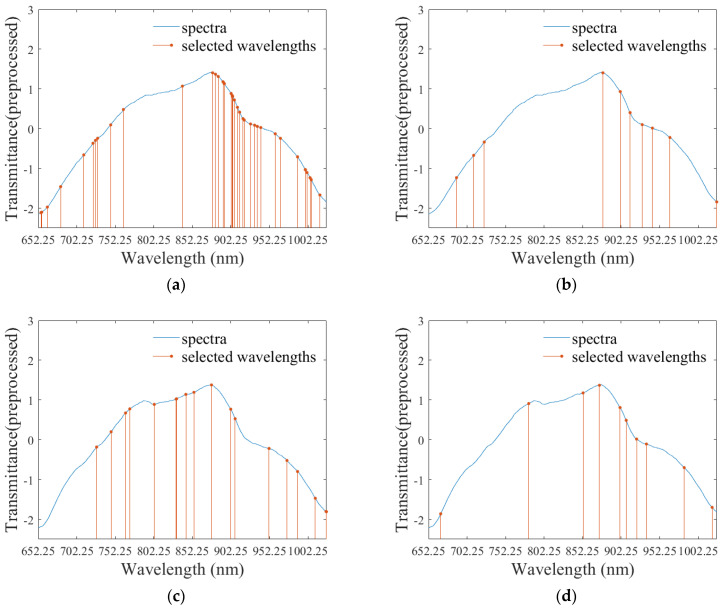
Presentation of the selected effective wavelengths: effective wavelengths selected by CARS for Orientation1 (**a**) and Orientation2 (**c**); effective wavelengths selected by SPA for Orientation1 (**b**) and Orientation2 (**d**).

**Figure 5:**
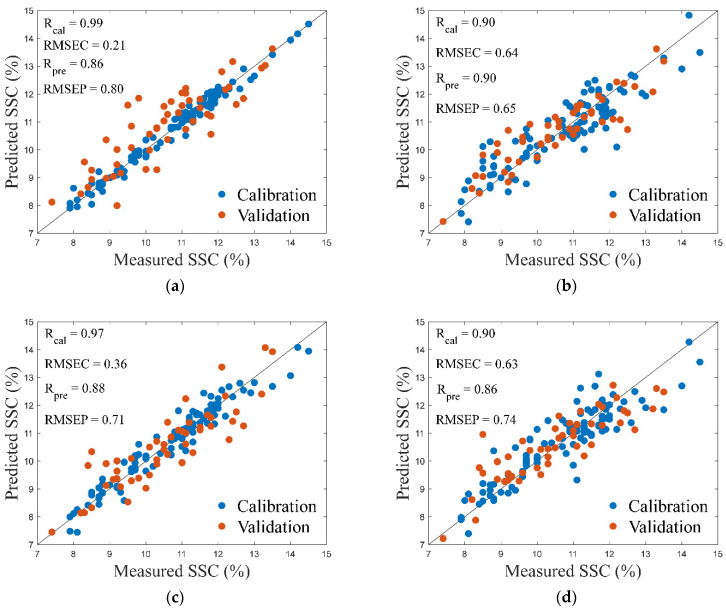
Scatter diagrams of evaluation results for models that were built using effective wavelengths: effective wavelengths selected by CARS for Orientation1 (**a**) and Orientation2 (**c**); effective wavelengths selected by SPA for Orientation1 (**b**) and Orientation2 (**d**).

Evaluation results for the models built by the wavelengths selected by CARS and SPA are shown in Table 4. For Orientation1, $R_{C}$, RMSEC, $R_{P}$, and RMSEP of the model built using the wavelengths selected by SPA are 0.90, 0.64%, 0.90, and 0.65%, and the corresponding values for Orientation2 are 0.90, 0.63%, 0.86, and 0.74%, both respectively. Figure 4 shows the wavelengths selected by CARS and SPA for both Orientation1 and Orientation2. Scatter diagrams in Figure 5 show the evaluation results in Table 4.

The figures clearly demonstrate that the models built with the wavelengths selected by SPA have more stable performance and less overfitting because there is less difference between calibration and validation in SPA-based models. $R_{P}$ and RMSEP are more approximate to $R_{C}$ and RMSEC. Additionally, SPA selects fewer wavelengths, significantly reducing the amount of calculation in the process of SSC prediction and suggesting that SPA is more efficient. Therefore, SPA is the more effective wavelength selection algorithm. The model in Figure 4b is the optimal SSC prediction model.

## 4. Conclusions

In this study, full transmittance spectra of peach fruit were collected at different orientations (Orientation1 and Orientation2). Then, the effects of spectrum-collection orientation and spectrum-collection region on the performance of online SSC prediction models for peach fruit were discussed. Average moving smooth combined with SNV was applied for preprocessing. The optimal selections of both orientations and regions were discussed. The results suggested that both Orientation1 and Orientation2 were ideal for spectrum collection and the S1-S3 was the better spectrum-collection region. Lastly, the effective wavelengths were selected from the final mean spectrum of S1-S3 by applying CARS and SPA. According to the results, wavelengths selected by SPA were more suitable for building the optimal online SSC prediction model of peach fruit. As a result, the developed model built using the full transmittance spectra has the potential for online SSC prediction. In terms of practical application, the developed model would simplify the feed mechanisms and fruit cups, decreasing the costs of detection. In addition, the discussion about spectra collection region in this study will be beneficial for improving the performance of the prediction model, enhancing the market competitiveness of the detection scheme.

Considering the robustness of the online SSC prediction model, the factors that were referred to above need further independent validation to confirm the relationships between them and SSC in peach fruit. Moreover, efficient model transfer technologies are necessary for actual use and for eliminating the adverse effects of different instruments, times, etc. such as in [36]. In addition, more factors that will affect the accuracy of SSC prediction should be taken into consideration in subsequent studies.

## Figures and Tables

**Table 1:** Distributional property of measured SSC values.

Data Set	Number of Samples	Min/(%)	Max/(%)	Mean/(%)	Std/(%)
Calibration	100	7.40	14.50	10.70	1.54
Validation	50	7.40	13.5	10.53	1.47

**Table 2:** Evaluating results of the models that were built with the final mean spectra for the different combinations of different parts of peach fruit at both Orientation1 and Orientation2.

Orientation	Combination	LVs	Rcv	RMSECV	Rc	RMSEC	Rp	RMSEP
Vertical	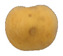	7	0.88	0.70	0.93	0.55	0.89	0.69
	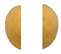	7	0.88	0.71	0.93	0.56	0.90	0.65
	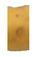	8	0.80	0.90	0.96	0.43	0.78	0.92
Horizontal	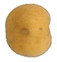	8	0.91	0.63	0.95	0.48	0.89	0.67
	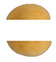	8	0.90	0.66	0.95	0.45	0.84	0.80
	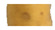	8	0.81	0.87	0.95	0.48	0.74	1.02

**Table 3:** Results for the selected effective wavelengths.

Orientation	Combination	Algorithm	Selected Effective Wavelengths (nm)
Vertical	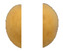	CARS	655.75, 656.75, 664, 681.25, 711.25, 723.25, 726.5, 729.25, 746.25, 762.75, 839.25, 878.5, 882.25, 885.75, 892, 892.5, 893.75, 902.5, 904, 904.5, 906.75, 910.75, 913.5, 918, 919.5, 927.75, 933, 936.5, 941, 959.75, 966.5, 988.75, 998.75, 999, 1001, 1005, 1006.5, 1017.75
		SPA	688.5, 710.75, 724.5, 878.5, 901, 913.75, 929.5, 942.75, 965.5, 1026.25
Horizontal	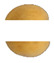	CARS	728, 746.75, 765.5, 771, 802.75, 831.25, 831.75, 843.75, 854.25, 877, 902, 907.75, 951.75, 975, 988.75, 1011.75, 1025.75, 1026.25
		SPA	668, 782.25, 852.75, 874, 900.75, 908.75, 922.25, 935, 984, 1020.25

**Table 4:** Evaluation results for the models that were built using the wavelengths selected by CARS and by SPA for both Orientation1 and Orientation2.

Orientation	Combination	Algorithm	LVs	Rcv	RMSECV	Rc	RMSEC	Rp	RMSEP
Vertical	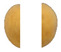	CARS	12	0.97	0.35	0.99	0.21	0.86	0.80
		SPA	8	0.88	0.70	0.90	0.64	0.90	0.65
Horizontal	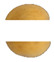	CARS	10	0.96	0.44	0.97	0.36	0.88	0.71
		SPA	7	0.88	0.70	0.90	0.63	0.86	0.74

## Data Availability

The data presented in this study are available on request from the corresponding author.
